# How Do Healthcare Professionals Develop the Communication Process to Promote Patients’ Health Literacy?

**DOI:** 10.3390/ijerph21050536

**Published:** 2024-04-25

**Authors:** Flaviane Cristina Rocha Cesar, Lizete Malagoni de Almeida Cavalcante Oliveira, Mariana Cristyen Galvão, Ana Luiza Andrade Lacerda

**Affiliations:** 1 Medical College, Mineiros University Center (UNFIMES), Mineiros 75833-130, Brazil; marianacristyengalvao@academico.unifimes.edu.br (M.C.G.); analuizalacerdaa03@academico.unifimes.edu.br (A.L.A.L.); 2 Nursing School, Federal University of Goiás (UFG), Goiânia 74690-900, Brazil; lizete@ufg.br

**Keywords:** health literacy, health communication, healthcare personnel, health education, health educators, consumer health information, patient education

## Abstract

This study aimed to analyze the communication process of healthcare professionals for the promotion of health literacy. It is a qualitative study that utilized individual online interviews with 46 healthcare professionals working in Brazil. The thematic content analysis technique proposed by Bardin was employed, and Atlas Ti software assisted in the assessment and interpretation of the texts. Content categorization revealed 26 sub-themes, and the coding of these identified nine themes and three categories. The communication process in health literacy education was composed of a set of interdependent and interrelated variables termed emotions, professional preparedness, interprofessional collaboration, patient needs assessment, building rapport, family inclusion in the educational process, environmental aspects, strategies, and resources for teaching and learning. These data may support the analysis of health communication in healthcare services, the creation of data collection instruments, and the development of training programs to enhance skills within the context of the identified variables.

## 1. Introduction

Health literacy (HL) can be defined as individual knowledge and skills developed throughout life that enable people to access, understand, evaluate, and use health information and services in a way that promotes and maintains their health and well-being [1]. The practice of HL includes experience, competence, or the use of HL skills in care [2]. To carry out this practice, healthcare professionals (HCPs) need to mobilize internal resources such as the knowledge of what to do, how to do it, and when to do it, building mental operations that result in skills for concrete actions and attitudinal, relational, and emotional aspects [3].

The process by which HL practices occur in healthcare services results from the interaction between patients, caregivers, and family members with HCPs. In this context, the ability to communicate assertively becomes essential, so HCPs must pay attention to the content (what is said), process (how it is said), and perception (thoughts, emotions, and difficulties) [4]. These components of health communication support the need for it to be clear, culturally sensitive, and appropriate to the diversity of HL in the population [5].

HCPs may have insufficient knowledge and practice in HL and communication to meet the information needs of patients, considering that the patient’s HL may initially be very weak, affecting even the most appropriate health communication skills [6]. The difficulty in adequately determining patients’ HL can lead to communication problems and can consequently compromise the safety and quality of care provided, for example, when the patient is unable to follow appropriate medical recommendations [7]. However, in the literature, there is a recurrent association of health professionals with the patient information and education process [8], suggesting that professionals can carry out practices that promote HL even without knowing its formal concept or term. Knowing these practices can allow us to understand how health professionals use communication as a strategy to promote HL.

Patients’ perspectives on how HCPs communicate seem to indicate significant issues. Patients experience a lack of communication, feelings of abandonment, and an inability to understand the information conveyed by HCPs in primary care services [9].

Patients with inadequate HL listed the essential skills of healthcare professionals for person-centered care, such as showing respect and understanding, using a comprehensible communication style, and involving patients in decision-making according to their needs [10]. Despite being active agents in actions for patient HL, the literature is still incipient in demonstrating how HCPs provide information to their patients in the context of HL and communication.

Describing the experiences of HCPs in the patient-informing process can identify difficulties, potentialities, and guide training to develop HL and communication skills. Additionally, it allows evaluating from the perspective of healthcare professionals how these actions have been carried out. Thus, this study aimed to analyze the communication process of HCPs for the promotion of HL. To achieve this goal, this study will analyze the experience of HCPs, such as doctors, nurses, dentists, and other graduated professionals working in primary care or hospital services, considering that these services have a high continuous flow of patients.

The results of this research can be valuable for identifying critical points in the training of healthcare professionals that may compromise communication with patients. In this regard, it is expected that healthcare services utilize our findings to conduct training for the healthcare team, thereby improving patient safety and the efficiency of patient education. Ultimately, we hope that the results of this study can help identify barriers and facilitators in the communication process to enable the development of health policies that promote HL.

## 2. Materials and Methods

This is a qualitative study based on the content analysis technique proposed by Laurence Bardin [11]. Qualitative research was chosen for this study to allow for the exploration of healthcare professionals’ experiences, considering it is a subjective phenomenon. On the other hand, content analysis was selected for data treatment due to its incorporation of a set of analysis techniques that enables the systematic and objective description of the content of messages (verbal or non-verbal), allowing inferences to be made about the conditions of the production/reception of these messages [11]. Thus, content analysis was employed to generate categories and facilitate the analysis of the relationship between the components identified in the communication process of the interviewees.

Participant recruitment was conducted through purposive sampling. Initially, HCPs who were authors of scientific publications identified through an uncontrolled search using the terms “Health Literacy” and “Primary Health Care” or “Hospital Care” with a filter for the Brazil region in the last five years (2015–2020) on Web of Science, PubMed, and LILACS, considered the main indexers of scientific journals in the health field, were invited by email.

A second recruitment strategy involved sending requests to Master’s and Ph.D. programs in the health field to share the research invitation with their students. These programs were identified in the georeferenced database of national postgraduate programs, covering all regions of the country [12].

Another strategy used for recruitment was sending email invitations to professionals recommended by participants. Upon completing the research form, participants were asked to indicate five more colleagues in the healthcare field, aiming to include all professional categories and regions of the country.

The participant recruitment process continued until all professionals from different categories and regions of the country were included, and no new categories were added to the dataset [13].

**Figure 1:**
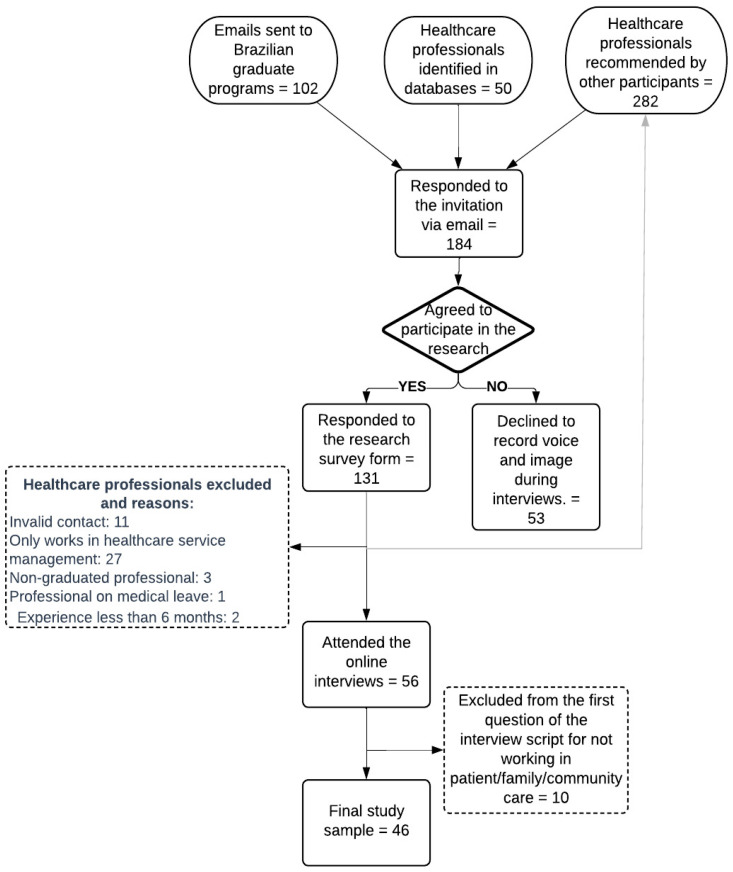
Construction of the study sample.

A total of 434 emails were sent, with a response rate of 42.4% (*n* = 184) from potentially eligible participants. Participants who indicated on the electronic form that they did not accept audio and video recording during the interview were considered refusals, totaling 28.8% (*n* = 53). It is noteworthy that out of the 87 invitations sent, 64.4% (*n* = 56) of participants attended the online interview (Figure 1).

Ten participants were excluded for not having a minimum of six months of direct patient care experience or not working in primary health care and/or hospital services. This period of experience in care can be considered a minimum to allow the opportunity to experience HL practices [14]. Therefore, 46 participants were included in the study sample.

### 2.1. Data Collection

The Data Were Collected in Two Phases from March to July 2020:

1. Online form—containing information about the research and Informed Consent Form (ICF) for the online interview. After being acquainted with the research details and the participation process, those who accepted the invitation were required to check the options “I agree to participate in the research” and “I allow the recording of my image/voice/opinion to be included in the research results”.
2. Individual online interviews—on the day and time previously scheduled with each participant, the researcher contacted them remotely (via email or WhatsApp^®^) and conducted the interviews (using the Google Meet^®^ application). The conversations were guided by a semi-structured script specially developed for this purpose, containing open-ended questions, such as “How do you provide health information to patients/families/caregivers?”; “What do you do when you perceive that the patient/family/caregivers have difficulty understanding or using the health information provided?”; and “How do you usually verify if patients/families/caregivers have understood the information provided?” The participants were instructed to answer the questions reflecting on their professional trajectory, not being limited by specific contexts such as COVID-19.

At the end of each interview, all participants were invited to add comments they deemed relevant about the experience of conveying health information and teaching about health to patients.

The interview script was previously submitted for content validation [15] with three experts in qualitative research and/or HL and underwent a pilot test with three individuals with characteristics similar to those of the study participants.

Each interview had its audio and video recorded and saved on an exclusive USB drive for the research, with a code for access and participant coding, allowing only the author to access the data and identify the participants.

### 2.2. Data Analysis

The content analysis technique proposed by Laurence Bardin [11] was used, and Atlas Ti software assisted in the assessment and interpretation of the texts. The analysis of the transcribed interviews was organized into three phases:

Pre-analysis: A project was created in Atlas Ti^®^, and the interviews were added as 46 PDF files. From this point, document groups were formed, and initial memos were written, indicating the relationship between the document and the research question, enabling the exploration and organization of the material. Initially, a floating reading of the transcriptions was conducted to identify relevant topics in the text composition. Subsequently, hypotheses and objectives for the research were defined, followed by the process of referencing the material. Key points were defined, indicating textual components for analysis, such as phrases and words. In this research, all quotes made by the interviewees regarding knowledge of, skills in, or attitudes about HL and/or facilitators of/barriers to HL practices were designated as key points.

Material exploration: Content categorization was performed by cutting the texts into record units. From these units, keywords were identified and categorized, resulting in initial categories through code creation and application in Atlas Ti^®^. Later, these were grouped thematically, resulting in intermediate categories, called subthemes, and grouped based on occurrences in themes, resulting in final categories. Text excerpts used to compose each theme were associated with a color code, allowing the identification of similar discourses. The open analysis model was used, allowing the constant reorganization of categories according to the insertion of new information that emerged during the process. Subthemes and themes were identified, generating codes and code groups, which underwent intense classification and reclassification work, resulting in analysis categories according to the principles of mutual exclusion (each element should not exist in more than one division), homogeneity within categories, relevance (reflecting research intentions), objectivity, and fidelity (understanding and clarity, avoiding distortions between coders), and productivity (providing fruitful results for inferences, new hypotheses, and accurate data).

The processing, inference, and interpretation of results: Initially, the transcriptions were subdivided according to the type of report to identify more clearly the knowledge, skills, and attitudes mentioned by the participants. Furthermore, the difficulties associated with HL practices were considered a report type. Data exploration was performed using tools, such as cluster items by word similarity and a word tree for knowledge, skills, and attitudes, to analyze relationships between expressions and words. Subsequently, an association was made between the unit of analysis (or citation content) and memos (observations recorded during collection). Afterward, the Atlas Ti^®^ analysis report was extracted in Excel format for reading and discussion among the study researchers.

Data quality was ensured considering the criteria proposed by Lincoln and Guba [16]: confirmability, credibility, dependability, and transferability.

### 2.3. Ethical Aspects

This study followed the principles contained in the Declaration of Helsinki and was approved by the Ethics Committee of the Federal University of Goiás, CAEE 17226919.1.0000.5083.

## 3. Results

The study sample comprised 46 HCPs from all regions of the country. Nurses (*n* = 16) and physicians (*n* = 13) constituted the majority of the study sample; 58.0% (*n* = 17) of the participants had ten or more years of experience in patient care, and the term HL had been heard by 41.3% (*n* = 19) and studied in-depth (during master’s or doctoral studies) by 8.6% (*n* = 4) of the participants (Table 1).

### Analysis of the Interviews

The pre-analysis of the interview transcription allowed the identification of 365 citation indexes, constituting the analysis corpus of the study. All citations involved the construction of informative health messages, the process of communicating with patients or family members, or professionals’ perceptions during HL education. Content categorization revealed 25 subthemes, and the coding of these enabled the identification of nine themes and three categories (Table 2).

Communication for HL education involved assessing the patient’s needs, considering their expectations, prior knowledge, and cognitive ability, as well as addressing emotional demands. Health professionals reported that this step guides the adaptation of teaching strategies to meet patient needs, a fundamental aspect for the effectiveness of educational actions. In this context, professionals’ preparation to develop competencies in educational activities preceded good practices in patient education.

Building a connection with patients and their families created a favorable scenario for adherence to provide guidance. Social aspects, cultural aspects, and the level of care (primary or tertiary) can become hindering factors influencing communication and the application of acquired knowledge.

Strategies employed by health professionals involved interactive methods with the sharing of experiences and group activities, as well as verbal communication. Assessing adherence to guidance was a frequent strategy reported by participants to allow adjustments in teaching strategies.

Resources used in educational activities were categorized into written materials such as brochures, posters, pamphlets, and manuals; information and communication technology (ICT) such as apps, social networks, and websites; and macro models. It is noteworthy that written materials were the most cited by respondents, highlighting the participants’ preference for this resource. Additionally, the incorporation of ICT was mentioned as a promising resource for activities with patients (Table 2).

**Figure 2:**
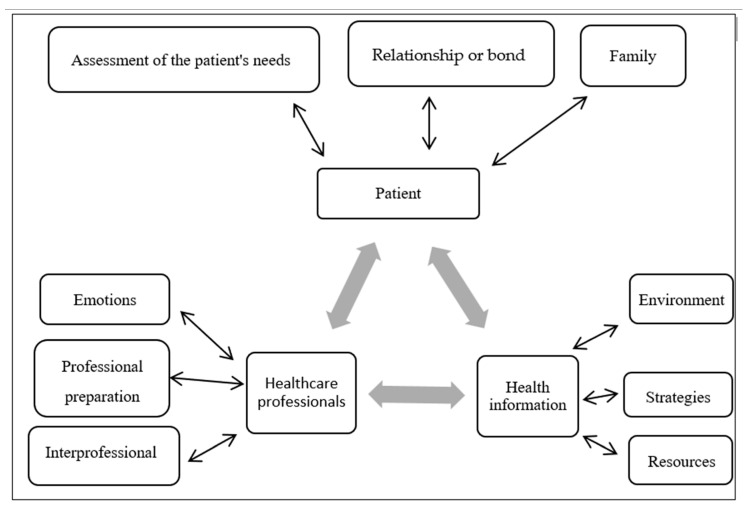
Illustration of the communication process in health literacy education, built from the experiences of health professionals. Source: constructed by the authors through the adaptation of the transactional and constructivist model of health communication proposed by Veenker and Paans [17] based on Steenbeek, Van Geert, and Van Dijk [18].

The communication process for HL education reported by the participants has at least three components: the patient, the health professional, and health information, and can be represented by a triangle with bidirectional relationships (Figure 2).

## 4. Discussion

This qualitative study aimed to analyze the communication process of HCPs for HL promotion based on their experiences and perceptions. To achieve this, a semi-structured interview analysis was conducted guided by Bardin’s content analysis. This analysis enabled the identification of the interviewees’ perceptions regarding the themes that characterize the communicative process in HL education.

The results of this study demonstrated that three main components represent the communication process in HL education: the patient, the HCPs, and health information, as observed in Table 2. This process aligns with the transactional and constructivist model of health communication [17]. This model assumes that learning occurs in a bidirectional process, where the patient (similar to a student’s role) learns and can assist the HCPs (similar to a teacher’s role) in improving their communication and teaching skills in HL [18]. This model is in line with a health communication perspective that transcends the linear sender–message–receiver (SMR) logic, as it considers the interaction between its components in a bidirectional manner.

The SMR logic focuses on assessing understanding and attitude change, considering that greater understanding enhances the ability for self-care [17]. On the other hand, the constructivist model focuses on communication as a support for patient autonomy, where the patient becomes an active agent in their self-care, and the HCP is a facilitator. This premise is supported by the self-determination theory, which argues that patient autonomy results from a process of adaptation and self-management [19]. In this sense, our results suggest that communication occurs bidirectionally, where the exchange of information between HCPs and patients is dynamic and non-hierarchical, as observed in Figure 1.

In our study, we emphasized that assessing patients’ needs, such as expectations, knowledge, cognitive abilities, and cultural aspects, can allow for better adaptation of communication to promote HL. The theoretical framework of the HL model for adult learners explains this importance [20]. This model presents sociocultural factors that can influence the translation of knowledge into health behaviors, including family history, ethnicity and culture, social, community, and work context, reading and health comprehension skills, and the information environment, such as financial issues, work, social activity, and the local community [20]. All of these aspects resemble those found in our study and reinforce that without a sociocultural assessment of the patient, there is a higher risk of communication that does not promote HL.

Our results showed that HCPs need to develop skills to engage in communicative processes that promote HL. Notably, there is a need for competence in managing their own emotions during the communication process, such as frustration when a patient does not understand a topic or does not achieve an expected result. On the other hand, HCPs also need to be able to identify negative feelings in patients that may compromise communication, such as fear, anger, or denial of a diagnosis. This aspect had not been mentioned in previous studies on professional skills in HL and may represent an advancement in professionals’ perception of emotional aspects of patient communication [21,22,23]. Additionally, data collection was conducted during the pandemic, where many people struggled with health information [24]. This may have contributed to sensitizing HCPs to the emotional aspects of health communication.

The HCPs interviewed in our study reported that assessing the patient’s emotional state allows for addressing urgent demands that, if unmet, could compromise the educational process. A previous study highlighted that difficulties in understanding information may be linked to emotional barriers, such as dealing with shock after a diagnosis. Strategies to overcome these barriers include having another person present during the appointment and bringing notes and/or questions to the consultation [25].

Regarding the competencies of HCPs in HL, our study also highlighted the need for professional preparation or training focused on developing theoretical and practical skills in health education. A recent literature review showed that professionals who receive training aimed at developing HL skills have more efficient communication and enhance patient autonomy in self-care [26]. Despite this, few curricular models in HCPs’ education support the development of communication skills for patient autonomy [17]. Therefore, although the interviewed participants recognized the importance of professional preparation for HL education, this remains a global challenge [27].

Finally, the last component of the communication process for HL promotion highlighted in our study was health information. In this component, study participants described factors that can interfere with health communication related to the environment, educational strategies, and resources used in the transmission of health information. A possible explanation for our interviewees identifying these factors is that communication depends greatly on how information is conveyed, including the amount of content, teaching strategy, and resource availability, as evidenced in other studies. Previous studies suggested, for example, not covering more than five topics in a single session, and participatory methods may be more effective for patient learning [5,28].

### 4.1. Limitations and Strengths of the Study

The study addressed the experiences of health professionals from different categories regarding HL education, allowing an understanding of this process from different epistemes. However, it is still necessary to consider the perspectives of the patients and their families. The qualitative approach allowed proposing the adaptation of an existing theoretical model to understand HL education; however, additional studies should verify the relationship of identified categories in different contexts. Another limitation of the study was the lack of a specific focus on the operational aspects of HL, such as the development of written and digital materials and the use of teach-back. These aspects were mentioned spontaneously by some interviewees, but it cannot be stated whether others use the fundamentals of HL appropriately.

### 4.2. Implications for Future Research

This study was innovative by exploring emotions in the communication process within the context of HL. This may influence further studies investigating interventions with patients and training for healthcare professionals in this context. The categories and themes identified in the study provide a snapshot of health education actions carried out in clinical practice. Consequently, the results of this study can support the development of training for healthcare professionals and care protocols to ensure that health education actions effectively promote critical HL among patients and the community. This study contributes to healthcare systems by describing how the communication process can be facilitated, potentially reducing healthcare costs, improving the quality of life for the population, and enhancing the efficiency of healthcare services.

## 5. Conclusions

Communication is a key precursor to developing patients’ HL. This study has highlighted that the communication experience for HL education constitutes a process of dynamic and reciprocal interactions between HCPs and patients and between them and health information. This process consists of a set of interdependent and interrelated variables related to professionals and patients, such as preparedness for health education; emotional aspects; and interprofessional collaboration. The variables related to the educational process itself include an assessment of patient needs; building individual and family bonds; environmental aspects; and strategies and resources for teaching and learning.

## Figures and Tables

**Table 1:** Sociodemographic characteristics of study participants (*n* = 46).

Variables		*n* (%)
Occupational category	Nurse	16 (34.8)
	Doctor/Physician	13 (28.2)
	Dentist	5 (10.9)
	Psychologist	5 (10.9)
	Nutritionist	4 (8.7)
	Physical Therapist	3 (6.5)
Gender	Female	30 (65.2)
	Male	16 (34.8)
	Non-binary	0 (0.0)
Age (in years)	≤30	11 (23.9)
	From 31 to 40	23 (50.0)
	From 41 to 50	8 (17.4)
	≥51	4 (8.7)
Duration of experience (in years)	≤5	10 (21.7)
	From 5 to 9	9 (19.6)
	From 10 to 14	10 (21.7)
	≥15	17 (37.0)
Academic level	Bachelor’s degree	4 (8.7)
	Specialization	15 (32.6)
	Master’s Degree	16 (34.8)
	Doctoral Degree	11 (23.9)
Level of health care in which you operate	Primary care	8 (17.4)
	Inpatient/Ambulatory Care	35 (76.1)
	Both	3 (6.5)
Region of the country in which you work	North	8 (17.4)
	Northeast	8 (17.4)
	Midwest	16 (34.8)
	Southeast	7 (15.2)
	South	7 (15.2)
Type of service you work for	Public	20 (43.5)
	Private	14 (30.4)
	Both	12 (26.1)

**Table 2:** Categories, themes, and speeches representative of the experiences of health professionals in health literacy education.

Category	Theme	Subtheme	Representative Speech
Healthcare professional	Emotions	Identify the professional’s feelings	[…] When we see that the person could improve, but is not following (the guidelines), it distresses us a lot. So we have to, sometimes, put our feelings aside a little […] (Physician 1)
		Identify the patient’s feelings	She was going through a phase of shock, of diagnosis, so she wasn’t elaborating on that disease situation itself. […] We simply listened, because sometimes the person had a demand that needed to be addressed before learning. (Nurse 1)
	Professional preparation	Develop competencies for health education	So, he has to have scientific knowledge, technical knowledge, he has to be prepared. He has to have skills, knowledge (Nurse 5)
	Interprofessional collaboration	Discuss with the team	You have to repeat. Call the social service and schedule a conference, say that we are going to discuss the case in a visit with a multidisciplinary team or our team, and that we will return to reinforce it. (Physician 2)
		Adapt to guidelines for using plain language	[…] So we always try to mediate between what is said by the technical team, both doctors and nurses, about care, and bring it to their reality […] (Psychologist 1)
Patient	Assessment of patient needs for health literacy	Understand customer expectations	Then I use this person-centered clinical method, which is to try to understand precisely what the expectations are in relation to their problem in order to be able to better explain […] (Physician 3)
		Consider prior knowledge of the patient	Before I offer any kind of information, I first raise what they already know and what they already have of that so that I can, on top of that learning they have, build a new one. (Psychologist 2)
		Assess cognitive ability	[…] That patient has some question even some alteration of consciousness there, maybe I need to leave it for another time […]. (Psychologist 3)
		Identify cultural aspects	So we start to learn–I didn’t graduate here, but there’s a language of its own here–[…] and then we try to bring this to the context of the subject, and then I learned here, for example, that the pupil, for them, is the apple of their eye […] (Psychologist 1)
	Relationship or bond	Provide bonding with the patient through communication	In fact, my way of informing patients is this, you have to look them in the eye, create a bond, create a relationship and inform. (Physician 4)
		Build bonding with family	So as we have more bonds with this family, the more bonds we have, the easier it is for us to walk. (Psychologist 1)
	Family	Involve a companion or family member in the educational process	There are cases like this, where I ask for a companion. Because there really are patients that I explain once, I explain twice, I explain three, four, five and they don’t understand, they do it wrong and then I ask them to call a companion. (Nurse 2)
Health Information	Environment	Identify barriers to the implementation of health information	Then, for example, what happens a lot: I have to admit patients because there is no way to do the proper treatment at home and, sometimes, we end up with a lot of social hospitalization, because we have to hospitalize, for example, a diabetic because she can’t afford the minimum. (Physician 5)
		Carry out educational activities in the hospital service or primary care	In tertiary care, it is more punctual. You’re not always with the patient on site, let’s say. In primary care, it was easier to use means, so we had the computer screen, for example […] in tertiary care, no, this information is verbal. (Nurse 3)
	Strategy	Promote the exchange of experiences between patients	So sometimes to prepare for a tracheostomy, I try to have conversations with peers, so, for example, I always have another patient who already has a tracheostomy in the ICU and then I ask for permission from the family, I put them to talk […] (Psychologist 1)
		Instructional demonstration	The resources would be, for example, to brush we have a macro model, to demonstrate how to brush our teeth, but we don’t have educational folders. (Dentist 2)
	Strategy	Use illustration	I really like to use a piece of paper, draw, draw flowcharts, mind maps. (Psychologist 1)
		Use individual or group activities	When it was like this, individually, I used more the written part, for example, or visual aids. […] If it was a group, I would also try to do dynamics or activities that expressed what I was trying to convey, so that I could get to the message. (Nurse 5)
		Use verbal communication	Through the dialogue itself, through the patients’ own questions, when they come to ask questions, they turn to the nurse of the sector, in this case, when we are there, and then we, through contact, personally, clear these doubts. (Nurse 6)
		Tailor the teaching process	For example, we tried to make the video for the elderly with larger letters, with a more paused sound. (Nurse 5)
		Recognize report of non-comprehension	So you tell me now, in short, what you understood. When they make the summary and give me feedback, I realized that the important information was absorbed, assimilated, it’s good. (Psychologist 2)
		Assess adherence to the guidelines	[…] If I gave a lecture, for example, at the hospital here, with patients who are in the hospital, talking about hygiene and I realize that the behaviors related to hygiene have not improved […] it probably means that my information has not arrived, so I will need some other resource […] (Psychologist 3)
		Perform shared decision-making	Everything we do in outpatient medicine with the patient is a suggestion. So, I wouldn’t even use that term teaching. I would suggest and use the term shared decision. (Physician 1)
	Resources	Use written material	There’s the dialogue part, verbal communication. And through the didactic materials that I make available: a guideline manual […]. (Nutritionist 3)
		Use information and communication technology	So, I have a Youtube channel, then if you want, subscribe there as well. There’s Instagram, there’s Facebook, I created an institutional WhatsApp, so I try, in this way, to make a link with them. […] (Physician 6)

## Data Availability

The data presented in this study are openly available in the FIGSHARE repository at https://doi.org/10.6084/m9.figshare.24943446.v1 (accessed on 28 January 2024).
